# A Transdisciplinary Analysis of COVID-19 in Italy: The Most Affected Country in Europe

**DOI:** 10.3390/ijerph17249488

**Published:** 2020-12-18

**Authors:** Flaminia Ortenzi, Emiliano Albanese, Marta Fadda

**Affiliations:** 1 Global Studies Institute, University of Geneva, 1205 Geneva, Switzerland; 2 Institute of Public Health, Faculty of Biomedical Sciences, Università della Svizzera Italiana, 6900 Lugano, Switzerland; emiliano.albanese@usi.ch (E.A.); marta.fadda@usi.ch (M.F.)

**Keywords:** COVID-19, Italy, epidemiology, containment measures, economic impact, mathematical modelling, social impact, social media, lessons learned

## Abstract

As of 27 March 2020, 199 countries and territories and one international conveyance are affected by the COVID-19 pandemic. As of the same date, Italy represents the third country worldwide in total number of cases and the first one in total number of deaths. The purpose of this study is to analyse the Italian case and identify key problem questions and lessons learned from the Italian experience. The study initially provides a general overview of the country’s characteristics and health care system, followed by a detailed description of the Italian epidemiological picture regarding COVID-19. Afterwards, all non-pharmaceutical measures adopted by the Government against COVID-19 are presented in chronological order. The study explores some estimations of the economic impact of the epidemic, as well as its implications for society, lifestyle, and social media reactions. Finally, the study refers to two types of mathematical models to predict the evolution of the spread of COVID-19 disease. Having considered all of the above-mentioned aspects, some significant issues can be raised, including the following: (1) the available epidemiological data presents some gaps and potential biases; (2) mathematical models always come with high levels of uncertainty; (3) the high number of deaths should be interpreted in light of the national demographic context; and (4) the long-term management of the epidemic remains an open question. In conclusion, the Italian experience definitely highlights the importance of preparedness and early action, effective interventions and risk communication.

## 1. Introduction

Coronaviruses are a family of viruses which affect the respiratory tracts and can cause various illnesses with different levels of severity, from simple colds and pneumonia, to Severe Acute Respiratory Syndrome (SARS) [1]. SARS-CoV-2 is a new coronavirus, which causes COVID-19 disease [1]. The outbreak of COVID19 first started in the city of Wuhan, China, in December 2019, although some evidence suggests that a few cases were already present in November 2019 or earlier, COVID19 spread worldwide rapidly, and it turned into a pandemic, as officially declared by the World Health Organization on March 11, 2020 [1]. As of 27 March, 199 countries and territories were affected globally, together with one international conveyance (the “Diamond Princess” cruise ship harboured in Yokohama, Japan) [2].

Italy reported its first two cases of COVID-19 at the end of January 2020, and approximately a month later the epidemic spread quickly in the Italian population [1]. As of the 27 March, Italy reported 80,589 cases. It was the third country worldwide in terms of total number of cases, after the USA and China, despite its markedly smaller population size [2]. The Northern part of Italy was the most affected part of the country with Lombardia, Emilia-Romagna and Veneto regions had the highest number (and proportion) of cases [1]. On the one hand, Italy registered more than 10,000 recovered patients (with an overall recovery rate of 55.5%); on the other hand, it reported a total of 8215 deaths as of the 27 March, which represented the highest number worldwide [1,2]. Indeed, Italy was the first and the most affected country in Europe and its Government was the first Western Government to implement strong mitigation measures against the COVID-19 epidemic, including so called lockdown (i.e., compulsory sheltering of healthy persons to prevent exposure to contagion) from Northern Italy, and then extending it to the whole country [3]. The peak of the first wave of the epidemic was reached at the beginning of May. The death toll was dreadfully high in March through April 2020, reaching nearly 1000 deaths per day at its peak.

In most European countries including Italy, the number of COVID19 cases progressively decreased in summer, and so did the R0 (e.g., the average number of secondary cases caused by a single infectious individual). Accordingly, most personal (i.e., hygiene and face masks), physical distancing (i.e., cancellation of gatherings, working from home), and movement measures (i.e., travels and movement of individuals between and within regions) were progressively relapsed. Europe has faced the anticipated second wave of the COVID-19 epidemic at the beginning of October, with some relevant variations across countries. For example, compared with France, Germany, the UK, and Spain, the COVID19 attack rate and effective reproduction number were relatively lower and slower between August and mid-October [4]. However, as of 2 December, the total number of cases of COVID-19 registered in Italy was 1,601,554, of which 407,791 cases were registered in Lombardia [1]. The second wave of the pandemic hit the region as hard as the first.

The Italian case can provide some useful insights for the overall understanding of the pandemic, and analysing its epidemiological situation, as well as interventions taken and their expected impact (both in terms of disease spread and socio-economic consequences) may contribute to generate questions and hypotheses. Moreover, some key lessons learnt in the region may be useful to other countries and/or for the prevention and control of future epidemics. The purpose of this study was to analyse the Italian case and identify key problem questions and lessons learned from the Italian experience. The study initially provides a general overview of the country’ characteristics and health care system, followed by a detailed description of the Italian epidemiological picture regarding COVID-19 during the first wave.

## 2. Case Presentation

### 2.1. Geographic, Climatic, Demographic, Economic and Political Characteristics of the Country

Italy can be described as a boot-shaped peninsula in the Mediterranean Sea and its territory includes numerous islands, with the largest ones being Sicily and Sardinia [5]. It is organized in 20 regions, and has six bordering countries: Austria, France, the Vatican, San Marino, Slovenia and Switzerland [5]. According to UN 2020 data, Italy has a total population of approximately 60.5 million people with a median age of 47.3 years [6]. The population density is 206 people per km^2^ on a total land area of 294,140 km^2^, and almost 70% of the Italian population is urban [6]. The largest city is Rome, which is the capital of the country [6]. The total fertility rate is 1.3 live births per woman, and it has been declining since the 1970s [6]. On the contrary, life expectancy at birth has constantly been increasing since the 1950s and is currently 86.0 years for females and 81.9 years for males, which makes Italy the sixth country in the world in terms of life expectancy [6]. The crude mortality rate is approximately 10.7 deaths per 1000 people [7]. The mortality rates for infants and children under the age of five are respectively 2.2 and 2.6 per 1000 live births; both rates have been decreasing substantially since the 1950s [6].

Italy is classified as a high income country with a GDP of 1,943,835 trillion USD and a GDP growth rate of 1.6%, according to UN 2019 data [8]. The most important economic sector of the country is the service sector, with more than 70% of the total employed population working in this sector, followed by the industrial sector (26% of the employed population) and the agricultural sector (less than 4% of the employed population) [8,9]. The male component of the labour force is higher than the female (58.1% vs. 39.9%) and the unemployment rate is 10.4% of the labour force and has recently seen an increasing trend [8]. In terms of international trade, UN 2019 data reports higher exports of goods and services than imports [8].

Regarding the political system, Italy is a Constitutional Democratic Republic and the Head of State is the President of the Republic, currently Sergio Mattarella [10]. The Italian Constitution establishes a three-way division of power into legislative, executive and judicial power, which are in the hands of the bicameral Parliament, the Council of the Ministers and the judges, respectively [10]. The adoption of non-pharmaceutical measures to contain the COVID-19 epidemic is the responsibility of the Council of the Ministers, headed by the Prime Minister, currently Giuseppe Conte [10].

### 2.2. The Italian Health Care System: Characteristics and Ranking

The two key features of the Italian health care system are its high level of decentralisation and the provision of universal health coverage [11]. Regarding the former, Italy has a regionally-based National Health Service (NHS), meaning that each region has the duty of organising and delivering health care services through local health units and public and accredited private hospitals [11]. Nevertheless, responsibility for health services remains shared between the central Government and the regions. The State plays a crucial role in establishing the legislative framework for health care and defining the main principles and goals of the NHS [12]. In addition, through the Ministry of Health, the central Government manages tax revenues for health financing, defines the content of the core benefit package (“livelli essenziali di assistenza”, essential levels of care) and is in charge of the overall coordination of the system [11,12]. All Italian citizens and legal foreign residents are granted access to hospital and medical care, free at the point of delivery (universal health coverage) [11]. Since 1998, illegal immigrants can also receive free urgent and essential care [11].

According to UN data, in 2019 Italy spent 8.9% of its GDP on health care [8], which is lower than the EU average of 9.8% [11]. The benefit package includes a wide range of services that are covered by public spending (74%); nevertheless, out-of-pocket payments by households are still relatively high (24%), while private health insurance only accounts for a small percentage of the total expenditure (2%) [11]. Regarding the number of doctors per population, in 2017 Italy had 4.0 doctors/1000 population, which is higher than the EU average of 3.6; however, the number of medical doctors working in public hospitals and as general practitioners is decreasing and more than 50% of doctors are 55+ years old, which implies that Italy is at risk of experiencing future shortages [11]. As opposed to the number of doctors, in 2017the number of nurses in Italy was only 5.8/1000 population, which is much lower than the EU average of 8.5 [11].

According to the Bloomberg ranking methodology for the evaluation of health care systems, in 2019, the Italian health system was ranked fourth place, after Hong Kong, Singapore and Spain, while in 2018 it was ranked sixth [13]. The Bloomberg ranking system only takes into account countries with a minimum GDP per capita of USD 5000, a population of at least 5 million people, and life expectancy of over 70 years [13]. The final outcome is mainly determined by the following factors: life expectancy, and relative and absolute costs of the health system, which explains why the top ranking positions have been assigned to advanced countries with low health system costs and high life expectancy [13]. In terms of cost-effectiveness, the services provided by the Italian health care system rank higher than many other European and non-European countries [13].

### 2.3. The Italian Epidemiological Situation Regarding COVID-19

**Figure 1:**
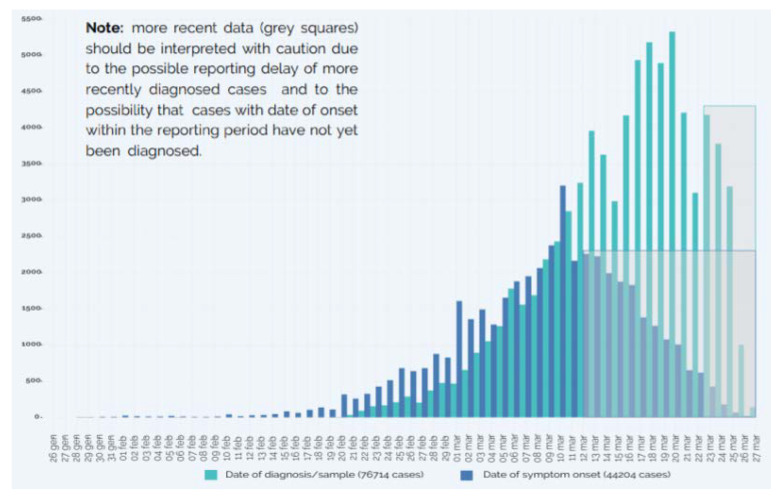
Number of COVID-19 cases by date of diagnosis/sample (*N* = 76,714) and number of COVID-19 cases by date of symptom onset (*N* = 44,204)—27 March 2020 [15].

Since the 28 February 2020, the Istituto Superiore di Sanità (ISS, “National Institute of Health”) has coordinated an integrated surveillance system that collects microbiological and epidemiological data provided by the Regions and the National Reference Laboratory (ISS) for SARS-CoV-2, and produced daily updated infographics and weekly bulletins on the evolution of the epidemic in the country [14]. In such infographics and bulletins, “cases” refer to all persons who have tested positive for SARS-COV-2 infection, based on qualitative detection of SARS-CoV-2 in upper and lower respiratory specimens with rt-PCR [14,15]. According to the bulletin published on the 19 March, 35,731 COVID-19 cases were detected and reported by regional laboratories and almost 99% of infection diagnoses in the samples sent from regional labs to the National Reference Laboratory were confirmed in subsequent swabs [14]. Of these, 3559 COVID-19 cases were detected among health care professionals (about 10% of all diagnosed cases), suggesting a potential high risk of infection transmission [14]. The total reported deaths were 3047 [14]. The number of new diagnoses saw an increasing trend from the end of February to the 19–20 March, whereas it appears to be decreasing afterwards, as shown by the infographics published on the 27 March (Figure 1) [15].

In terms of gender and age distribution, as of the 19 March, 59% of cases were male (20,686) and the median age was 63 years [14]. The case fatality rate (CFR) increased in older age groups, and all age groups showed higher lethality for males compared to females [14]. Approximately 69% of deceased individuals were affected by at least one co-morbidity, including cardiovascular diseases, respiratory diseases, diabetes, immune system deficiencies, metabolic diseases, cancer, obesity, kidney diseases or other chronic conditions [14]. As of the same date, the assessment of the clinical status of patients, available for 12,960 cases, produced the following results: 6.0% of cases were asymptomatic, 8.8% showed extremely mild symptoms, 11.0% had symptoms of unspecified severity, 45.4% presented mild symptoms, 24.0% had severe symptoms requiring hospitalization and 4.8% were very severe cases in need of intensive care [14].

**Figure 2:**
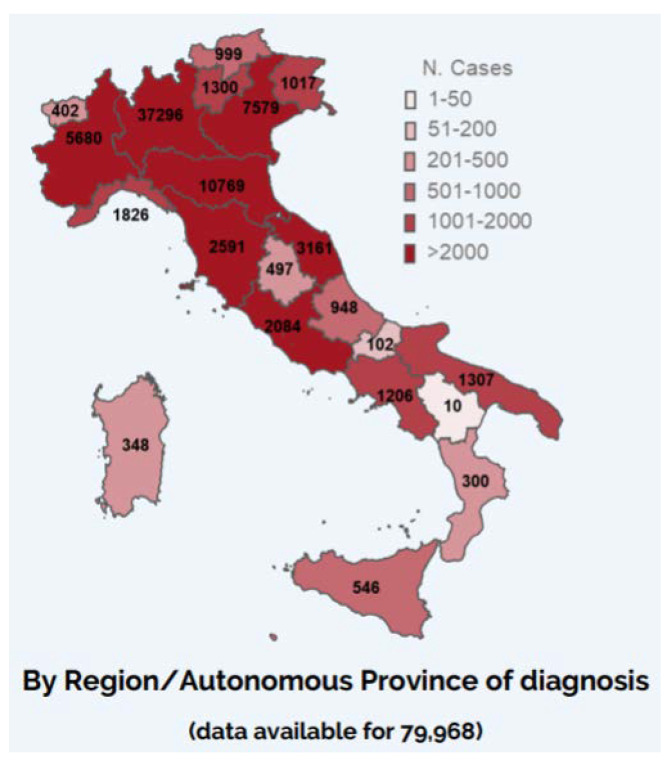
Number of COVID-19 cases diagnosed by Region/Autonomous Province of diagnosis (*N* = 79968)—27 March 2020 [15].

For what concerns the regional distribution of cases, as of the 19 March, COVID-19 cases had been reported in all Italian regions [14], with Lombardia (which represents the epicentre of the epidemic), Emilia Romagna, Veneto and Marche being the most affected ones, while in some other regions, cases were more sporadic, with more limited transmission chains [14]. As of the 27 March, Piemonte, Toscana and Lazio were added to the list of most affected regions (Figure 2) [15].

**Figure 3:**
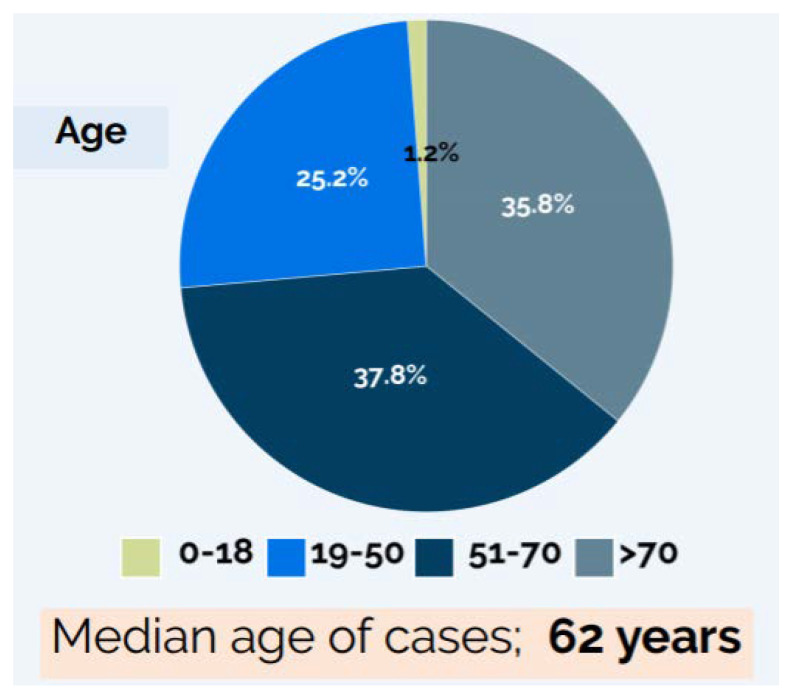
Age distribution of COVID-19 cases—27 March 2020 [15].

The epidemiological situation of the country has been constantly evolving until today. Indeed, the infographics of the 27 March showed a total number of 79,968 COVID-19 cases (of which 7145 cases among health care workers), with 7590 associated deaths [15]; while the bulletin of the 30 March reported a total of 94,312 cases and 10,026 related deaths [15]. However, the gender and age distribution of cases, as well as the age-specific CFRs, remained similar to those described in the 19 March bulletin (Figure 3 and Table 1) [15].

## 3. Management and Outcome

### 3.1. Non-Pharmaceutical Intervention Measures Taken by Italian Authorities

The first action on the COVID-19 epidemic taken by the Italian Government was the Decision of the Council of Ministers (31 January 2020) to declare the state of emergency with a six-month duration, and allocate Euro 500 million for the implementation of initial interventions [16]. A few days later, on the 3 February, the Ordinance of the Head of the Civil Protection Department was issued. The Ordinance stated that the Head of the Civil Protection Department was responsible for the coordination of several public and private actors involved in emergency management and relied on the expert opinion of a Technical and Scientific Committee [16].

The 23 February Decree Law (Urgent measures regarding the containment and management of the epidemiological COVID-19 emergency) established a “Red Zone” (11 Municipalities in Northern Italy), where a series of mitigation measures were adopted, including: prohibition of movement from, to and within the Red Zone; suspension of all public and private events and mass gatherings, and of all in-person educational (i.e., schools) and work/business activities; closure of all commercial activities, except from those providing essential services, which could only be accessed wearing masks, and enforcement of hand hygiene, physical distancing, and respiratory etiquette [16,17]. (The kind of PPE required is not explicitly mentioned in the 23 February Decree Law. Information related to Personal Protective Equipment to be worn when accessing essential commercial activities is now easily available on the website of the National Institute of Health (December 2020): mask wearing is mandatory and gloves should be used when selecting bulk products). Subsequently, the 1 March Decree Law (Further implementing provisions of the 23 February Decree Law) extended these measures to three regions (Emilia Romagna, Lombardia, Veneto) and two provinces (Pesaro-Urbino and Savona) at high risk; in addition, the Decree Law established the suspension of activities in most leisure facilities, whereas places of worship, restaurants, bars and other commercial activities were allowed to remain open provided that visitors observed the interpersonal safety distance of at least one metre [16].

The 8 March Decree Law (Further implementing provisions of the 23 February Decree Law) made a distinction between measures to be taken in the most affected regions and provinces of Northern Italy and interventions for the rest of the country [16]. The former included all restrictions already formulated in the previous Decree Law (1 March), in addition to some new ones including: prohibition of entry and exit and all non-essential movements within these areas; recommended self-quarantine in case of symptoms of respiratory infection and fever and mandatory quarantine for confirmed cases; closure on weekends of commercial activities, with the exception of drugstores, pharmacies and food outlets [16]. The latter also included all measures established by the 1 March Decree Law, plus the following: strict restrictions on relatives and visitors’ access to hospitals, residential structures for the elderly and other health care facilities; implementation of home working; isolation/quarantine of new prisoners if symptomatic, and existing prisoners that test positive [16,18]. The duration of such quarantine periods is not specified in the 8 March Decree Law. The circular issued by the Ministry of Health on the 12th of October 2020 (Ministry of Health Circular n. 32850, COVID19—directions for the duration and ending of isolation and quarantine), which remains valid to the present day (December 2020), recommends the following: 10 days of isolation starting from the day of test execution for confirmed cases and 14 days of quarantine for close contacts of COVID-19 cases without test execution (or 10 days of quarantine followed by test execution). The day after, the 9 March Decree Law (“#iorestoacasa”, #Istayathome) extended the interventions previously implemented only in the most affected areas to the whole national territory until the 3rd of April [16]. Subsequently, the 11 March Decree Law reinforced the previous Decree Law through the following measures: suspension of all commercial activities, with the exception of the provision of food, medications and other basic necessities; suspension of the activities of catering services with the exemption of home delivery services, whereas banking and insurance services and the agricultural, livestock and food processing sector remain functioning; public transport, automotive services, rail, air and sea transport are restricted to the minimum essential services [16].

Afterwards, the 22 March Decree Law imposed the closure of all non-essential production activities until the 3 April and the prohibition of movement for all individuals, except for proven work necessities, absolute urgency or health-related reasons [19]. The 24 March Decree Law listed a series of temporary containment measures that could be adopted for a set period of time in specific regions or the entire national territory, including: restriction of movement for all individuals, prohibition of movement for quarantined infected patients and precautionary quarantine for close contacts; suspension of leisure, sporting, cultural, social and religious events and activities, and of educational services and public transport; restriction or suspension of Government and commercial activities, without compromising essential services, and of all business, professional and self-employment activities [20].

### 3.2. Economic Impact and Mathematical Modelling Predictions

In terms of short-term economic impact, according to a forecast from March 2020, it was estimated that the Italian GDP will decrease as a result of the COVID-19 epidemic; specifically, the GDP was expected to drop by 3% by the end of the first quarter of 2020 and by 5% by the end of the second quarter of the year [1]. Regarding long-term economic impact, some forecasts showed that the country’s GDP will experience an overall −3% change in 2020 compared to 2019, and it will start growing again from 2021 [1]. On the contrary, according to other estimations, the Italian GDP will neither increase nor drop in 2020, showing a 0% change [1]. Moreover, both the domestic demand and the industrial production were forecasted to drop in 2020 compared to 2019 (by 2.8% and 4.6%, respectively), but they were believed to increase again starting from 2021 [1]. However, the impact of the novel coronavirus will not be the same on all economic sectors: the textile, transport, hotels, restaurants, sports and entertainment industries were expected to be the most highly affected, while the food, health, cosmetics, and media industries are actually expected to grow [1]. The tourism industry will undergo great economic losses, as it was believed that in 2020 Italy is likely to register a decrease of approximately 4.7 million international tourists’ arrivals, largely related to Chinese, German and USA tourists [1]. As a result, according to the forecasts from March 2020, the country’s complete lockdown lasting until April 2020 would result in approximately Euro 4 billion losses in terms of tourism value added [1]. Of course, predictions of COVID-19 economic impact should be evaluated cautiously and are subject to constant evolution and adjustments as new data is collected and analysed. For instance, according to some recent estimations (November 2020), the Italian GDP is expected to decrease by 9.9% in 2020 [1]; this percentage greatly differs from the ones provided in March 2020.

Recently, the Institute of Applied Mathematics of the Italian National Research Council (Cnr-Iac) in collaboration with the Imperial College of London have been analysing the spread of COVID-19 in Italy by applying different mathematical and statistical models to make predictions about the duration of the epidemic and the percentages of infected and deceased patients [21]. One of the existing approaches is based on geometric and logistical models (parametric models), which are commonly used to describe the evolution of epidemics; another approach consists in applying compartmental mathematical models, usually used in epidemiology [21]. Estimates obtained by taking the first approach and by analysing data up to the 16 March, predicted that the number of infected individuals will stabilise in the period between 25 March and 15 April [21]. Regarding the second approach, in addition to the traditional compartments of susceptible, infectious, recovered and deceased individuals, a new category has to be introduced to account for “healthy carriers” [21]. By analysing data up to the 16 March, a modest decrease was detected in terms of growth rate of the infectious compartment in Lombardia and it was predicted that a significant reduction would occur in a week’s time from that date as a result of the containment measures adopted [21]. Similar outcomes were predicted for the Central part of Italy. As opposed to the North, the South of Italy experienced an increase in the growth rate of the infectious compartment, probably due to an exodus from the North to the South following the 8 March Decree Law [21].

### 3.3. Impact on Society and Implications of the Media and Social Media

In terms of impact on society, the complete lockdown has pushed Italy towards a “digital revolution” [22]. In fact, the closure of schools, universities, business companies, leisure and commercial facilities has led to an unprecedented development of online education, smart working and home entertainment and has encouraged more and more consumers to shift to e-commerce and rely on online grocery shopping [22].

The COVID-19 epidemic has also caused social stigma and discriminatory behaviours against people from highly affected areas and people who might have been in contact with the virus [23]. The social stigma is mainly due to the fact that COVID-19 is a new disease with many unknown factors that generate confusion, fear and anxiety among the public, and it is easy to associate these negative feelings with “others” [23]. The consequences of stigma are the disruption of social cohesion and the isolation of some population groups, leading to the establishment of a social context which is actually more favourable for the spreading of the virus [23]. In fact, people are more likely not to report illness and not to seek health care immediately because they are afraid of discrimination and they can also be discouraged from adopting healthy behaviours [23]. Stigmatization and discrimination are highly associated with the “infodemic” of fake news and rumours about COVID-19, as it has been referred to by the WHO [23]. Therefore, the Italian Government, health authorities and the media play a key role in preventing stigma, as they are responsible for providing evidence-based, scientific facts through ethical journalism, adequate communication strategies (i.e., social media), and appropriate language [23].

By observing the evolution of the amount and the tone of conversations on social media throughout the course of the epidemic in Italy, it can be noticed that each peak corresponds to an event that had a strong impact on public opinion [24]. For instance, on the 24 March most conversations revolved around the new measures adopted with the 24 March Decree Law [24]. In addition, it is possible to analyse the most popular themes, phrases and words regarding COVID-19 that are being used on social media; some of them have constantly been present since the beginning of the epidemic, such as “intensive care” and expressions of criticism towards the Italian health system, which, in people’s perceptions, is about to collapse [24]. Of course, politicians and national authorities also avail themselves of social media as a means of political propaganda and communication, and their posts are mainly about new decree laws adopted [24]. Moreover, social media sentiment and emotion analysis revealed an ongoing slight increase in terms of optimistic attitude among social media users [24]. In fact, in the last few weeks, posts of solidarity and hope and positive emotions (i.e., admiration and joy) have started outnumbering messages of panic and deep uncertainty and negative emotions (i.e., anger and fear), which dominated the first weeks of the outbreak [24].

## 4. Discussion

Some gaps and key issues can be identified in the Italian case. First of all, there are some gaps and potential biases in epidemiological data. Increasing or decreasing trends in the number of new diagnoses are likely due to changes in testing policies, and may be biased. For instance, individuals with severe symptoms are more likely to be tested than those with no symptoms [25]. Next, common tests used for COVID-19 diagnosis may be not accurate, and their validity may be poor [26]. In addition, the date of symptom onset was not available for all diagnosed cases [18]. According to the National Institute of Health, this can be due to the fact that some of the diagnosed cases have not yet developed symptoms and/or due to the failure to consolidate the data itself. Moreover, the clinical status of positive cases was not available for all patients and was not assessed in a standardized way in all regions [14]. Such information and measurement biases can compromise the quality and completeness of data, which would greatly affect the accuracy and reliability of mathematical models and estimations of socio-economic impact of COVID-19. Caution is warranted in the interpretation of data.

Second, mathematical predictions about the future evolution of the epidemic are subject to a significant level of uncertainty due to several influencing factors, such as people’s individual behaviours and compliance with containment measures that are being implemented [21], meaning that it will take some time before it is possible to actually evaluate the efficacy of such interventions. Epidemiological investigations suggested that the infection transmission took place within the country for all identified cases, with the exception of the first two ones that were reported by the Lazio region and were probably infected in China [14]. Regarding the regional distribution of cases, by the 19 March, COVID-19 cases had been reported in all Italian regions [14]. Cases were mainly concentrated in the Northern part of the country, especially in the regions of Lombardia (which represents the epicentre of the epidemic), Emilia Romagna, Veneto and Marche [14]; however, according to the infographics published on the 27 March, Piemonte, Toscana and Lazio add to the list of most affected regions (Figure 2) [15]. In some regions, cases are more sporadic, with more limited transmission chains [14].

Third, the number of deaths in Italy has now surpassed China’s officially reported deaths [2], but when analysing this type of epidemiological data, the national context should always be taken into account: the Italian population is relatively old (median age = 47.3 years) [6] and the CFR for COVID-19 is higher for the elderly; also, social distancing is not part of the Italian culture, unlike other cultures where it is implemented in people’s daily routines [3]. Social distance has so far generated serious consequences, affecting social relationships and interactions (especially the empathic process), the economy and employment rates, and widening the digital and cultural infrastructure divide [27].

Last but not least, the most important question remains on how to manage the epidemic in the long-term in view of the need at some point to restore economic activities [3]. It has been argued that a strong and better economic recovery from the COVID-19 pandemic requires governments to make the services sector a key element in their policy mix, as services play a key role in increasing productivity, efficiency and effectiveness in the whole economy [28]. On 27th October, the Italian Government approved the “Ristori” Decree Law [29], which introduces further urgent measures for the protection of health and for the support of workers and production sectors, as well as in the field of justice and safety related to the COVID epidemic -19. The Decree Law allocated 5.4 billion euros in terms of net debt and 6.2 billion in terms of balance for the restoration of the economic activities concerned, directly or indirectly, by the restrictions placed on health protection by the 24th of October Decree Law. This Decree, valid until today, mandated that public places (bars and restaurants) may remain open every day including weekends and holidays until 6.00 pm; closure of gyms and swimming pools; and suspension of cinema and theatre activities [30].

## 5. Conclusions

In conclusion, since Italy is the first and the most affected country in Europe, some lessons can be learned from the Italian case which could be useful to other European and non-European countries. First of all, the government initially underestimated the potential impact of the new epidemic, by allowing people to get on with their normal lives, and overestimated the country’s preparedness to deal with it [3]. For instance, at the end of January 2020, the Prime Minister declared that Italy was fully prepared to respond to COVID-19, and, when Milan (Lombardia), which is the country’s financial capital, was first hit by the epidemic, the political class spread a video with the slogan “Milan does not stop” [3]. On the other hand (second lesson learned), it must be acknowledged that Italy was the first Western country to adopt very severe containment measures, which are apparently leading to early signs of improvement and are being used as a model by other countries [3] Indeed, as described above (Section 3.1), the government moved quickly from quarantining eleven municipalities in Northern Italy (“Red Zone”) to placing the whole national territory under lockdown and interrupting all non-essential economic activities. Third, a large number of health care professionals has been infected (as mentioned in Section 2.3), which means they might be overexposed to the risk of infection due to lack of adequate PPE and safe working conditions, which would allow them to perform their duties while at the same time preserving their health and not becoming potential transmission channels themselves [3]. Fourth, communication strategies have not always been efficient and have generated waves of public panic [3]. This appears particularly evident when considering the following example: the government’s decision to put some areas of Northern Italy under full lockdown was communicated to the press before being officially approved, which resulted in an exodus towards the South of the country and the subsequent spread of the virus (Section 3.2) [3]. As it has been argued that Italy wasted these lessons during the second wave of the pandemic [31], it is important that what is learned is then followed up with appropriate actions from governments and other stakeholders involved in the management of the pandemic.

## Figures and Tables

**Table 1:** Age and gender distribution of cases and deaths with age-specific CFRs—30 March 2020 [15].

Age Group	Male					Female					Total				
	*N*. Cases	% Cases by Sex	*N*. Deaths	% Deaths by Sex	% CFR	*N*. Cases	% Cases by Sex	*N*. Deaths	% Deaths by Sex	% CFR	*N*. Cases	% Cases by Age Group	*N*. Deaths	% Deaths by Age Group	% CFR
0–9	324	55.5	0	0.0	0.0	260	44.5	0	0.0	0.0	589	0.6	0	0.0	0.0
10–19	392	51.4	0	0.0	0.0	371	48.6	0	0.0	0.0	766	0.8	0	0.0	0.0
20–29	1631	43.3	1	50.0	0.1	2132	56.7	1	50.0	0.0	3830	4.1	2	0.0	0.1
30–39	3105	48.1	18	90.0	0.6	3352	51.9	2	10.0	0.1	6523	6.9	20	0.2	0.3
40–49	5802	48.4	66	74.2	1.1	6198	51.6	23	25.8	0.4	12,084	12.8	89	0.9	0.7
50–59	10,068	54.2	294	79.9	2.9	8495	45.8	74	20.1	0.9	18,678	19.8	369	3.7	2.0
60–69	10,744	65.8	923	79.6	8.6	5584	34.2	236	20.4	4.2	16,395	17.4	1162	11.6	7.1
70–79	11,236	64.7	2597	75.3	23.1	6142	35.3	854	24.7	13.9	17,464	18.5	3456	34.5	19.8
80–89	7630	54.0	2603	65.4	34.1	6504	46.0	1378	34.6	21.2	14,186	15.0	3984	39.7	28.1
>90	1160	32.5	424	45.2	36.6	2404	67.5	514	54.8	21.4	3573	3.8	939	9.4	26.3
Unknown	114	51.6	4	80.0	3.5	107	48.4	1	20.0	0.9	224	0.2	5	0.0	2.2
**Total**	52,206		6930		13.3	41,549		3083		7.4	94,312		10,026		10.6
